# Low-Cost Polyphenol–Polypyrrole Molecularly Imprinted Sensor for Point-of-Care Alzheimer’s Detection

**DOI:** 10.1021/acssensors.5c01816

**Published:** 2025-08-15

**Authors:** Ajith Mohan Arjun, Sudhaunsh Deshpande, Guoyi Liu, Daimei Miura, Krzysztof Pawlak, Tokuda Takahiko, Makoto Higuchi, Miyu Matsumoto, Tomoko Umemura, Kaori Tsukakoshi, Sanjiv Sharma

**Affiliations:** 1 David Price Evans Global Health and Infectious Diseases Group, Pharmacology & Therapeutics, Institute of Systems, Molecular and Integrative Biology, 4591 University of Liverpool, Crown Street, Liverpool L69 7BE, United Kingdom; 2 Key Laboratory of Optoelectronic Technology & Systems, Chongqing University, Chongqing 400044, China; 3 Department of Biotechnology and Life Science, Tokyo University of Agriculture and Technology, 2-24-16, Naka-cho, Koganei, Tokyo 184-8588, Japan; 4 Institute of Global Innovation Research, Tokyo University of Agriculture and Technology, 3-8-1 Harumi-cho, Fuchu, Tokyo 183-8538, Japan; 5 Materials Innovation Factory, 4591 University of Liverpool, 51 Oxford Street, Liverpool L7 3NY, United Kingdom; 6 Advanced Neuroimaging Center, Institute for Quantum Medical Science, National Institutes for Quantum Science and Technology (QST), 4-9-1 Anagawa, Inage-ku, Chiba-shi, Chiba 263-8555, Japan; 7 Department of Chemistry, Faculty of Science, Tokyo University of Science, 1-3 Kagurazaka, Shinjuku-ku, Tokyo 162-8601, Japan

**Keywords:** Alzheimer’s disease, point-of-care (POC) diagnostics, electrochemical biosensor, phosphorylated tau 181 (p-tau181), molecularly imprinted polymer (MIP), polyphenol red−polypyrrole (pPhR-pPy)

## Abstract

There is an urgent need for rapid, affordable diagnostics for Alzheimer’s disease (AD), particularly those that can detect phosphorylated Tau 181 (p-tau181)—a key biomarker—directly in plasma. We present a scalable, label-free electrochemical biosensor fabricated on a printed circuit board platform, integrating a novel nanomolecularly imprinted polymer transducer based on a polyphenol red–polypyrrole composite. This platform enables sensitive detection of p-tau181 in undiluted plasma, serum, and clinical AD samples using a custom-designed potentiostat and machine learning-assisted classification. The sensor achieves a detection limit of 498 fg mL^–1^, a sensitivity of 8.99 μA/pg mL, and an area under the ROC curve (AUC) of 0.84 in clinical samples, comparable to the single molecule array (SIMOA) assay (AUC = 0.91), but at a fraction of the cost. This integrated platform, validated in real patient samples, demonstrates significant advancement toward accessible, point-of-care diagnostics for early AD detection.

## Graphical abstract

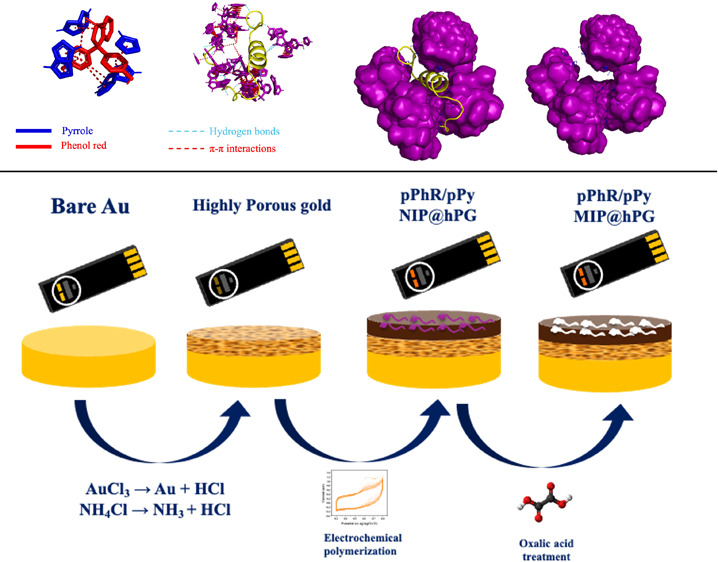

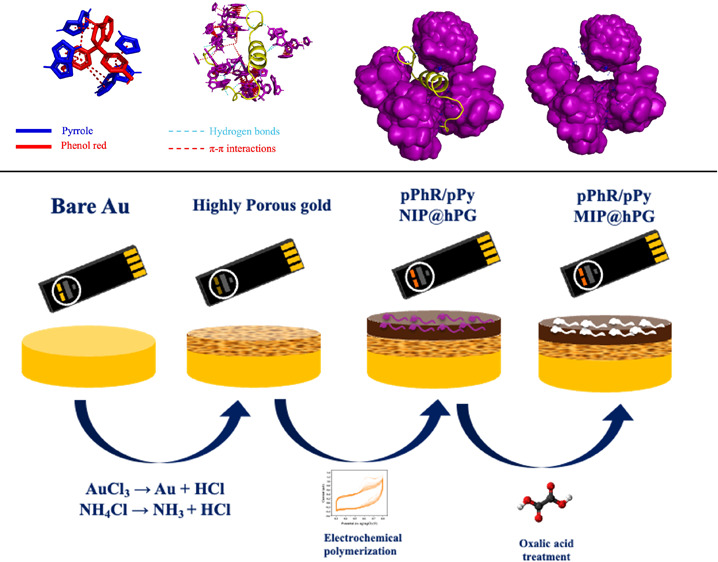

Alzheimer’s disease (AD) is the most common neurodegenerative disorder, currently affecting over 50 million people worldwide, with projected increases driven by population aging.^1^ In countries such as the UK and Japan, it is a leading cause of mortality.^2,3^ Early diagnosis is crucial for initiating therapeutic interventions, managing symptoms, and improving patient outcomes.^4^ However, current diagnostic methods, including magnetic resonance imaging (MRI), computed tomography (CT) scans, fluorodeoxyglucose positron emission tomography (FDG-PET), amyloid PET, Tau PET imaging, clinical assessments like Mini-Cog, and tests like immunoassays (ELISA or SIMOA), are costly and time-consuming and require specialized equipment and expertise, limiting their accessibility.^5–9^ The need for rapid, sensitive, and cost-effective diagnostic alternatives is therefore paramount, particularly point-of-care (PoC) biosensors that can triage patients efficiently, reduce clinical workload, and enable early intervention.

Electrochemical biosensors offer a promising PoC solution, providing high sensitivity, rapid response times, and miniaturization potential.^10^ While optical biosensors including colorimetric, fluorometric, and surface plasmon resonance-based assays are widely used, they often suffer from spectral crosstalk, photobleaching, and interference from endogenous chromophores.^11–14^ In contrast, electrochemical biosensors transduce recognition events into electrical signals, facilitating real-time, label-free detection.^15^ Conventional electrochemical biosensors rely on enzymes, antibodies, and aptamers for target recognition, but these bioreceptors pose challenges in terms of stability, storage, and reproducibility.^16–20^

To address these limitations, nanomolecularly imprinted polymers (nMIPs) have emerged as synthetic receptor analogues with high specificity, chemical stability, and cost-effectiveness.^21–26^ The redox-active properties of specific polymers can further enhance sensor performance, eliminating the need for additional enzymatic or fluorescent labels.^27,28^ Polyphenol red (pPhR), a redox-active mediator, has been previously utilized in enzyme-coupled systems for zinc and drug detection.^29–33^ However, its susceptibility to degradation under light, heat, and pH variations, as well as its intrinsic nonconductivity, limits its standalone use in biosensing applications.^34,35^

To overcome these challenges, we introduced a polyphenol red–polypyrrole (pPhR-pPy) composite. This composite leverages the advantageous properties of polypyrrole (pPy), including its electrical conductivity, mechanical robustness, and substrate adhesion.^36–38^ The conjugated system of double bonds in pPy facilitates electron delocalization; combining it with pPhR is expected to further increase this delocalization and overall conductivity, enhancing signal transduction.^39^ Furthermore, the improved flexibility and toughness from pPy can improve the structural integrity and functionality of a sensor based on the pPhR-pPy copolymer. The redox activity of this composite polymer, observed in the range of 0.3–0.5 V, combined with the possibility of electropolymerizing pPhR on electroactive surfaces makes it a promising material for developing redox-active MIP-based surfaces. This approach uniquely combines the redox mediation of pPhR with the conductive and mechanical properties of pPy, enabling label-free detection without external redox probes. In this report, we present an nMIP-based sensing surface composed of this composite of polyphenol red and polypyrrole (pPhR-pPy).

To realize the practical application of these pPhR-pPy nMIPs in a scalable sensing platform, we integrated them into cost-effective printed circuit board (PCB)-based electrodes. The well-established infrastructure in PCB manufacturing for high-volume production translates to significant cost and volume advantages for those systems where the objective is to scale up. A further amplification is presented by reproducibility of the PCB fabrication process. While the high-volume production and reproducibility of PCB manufacturing offer significant advantages, a critical challenge arises from the standard surface finish, electroless nickel immersion gold (ENIG). This method involves the use of a thin layer of gold over the nickel, which can be easily damaged or worn away, which exposes the underlying nickel.^40^ The presence of nickel leads to unstable signals and a lack of reproducibility among the different electrodes. To mitigate this problem, we employed a technique for the deposition of gold over the ENIG using a gold brush plating solution. The use of this gold surface has resulted in reproducible signals corresponding to the presence of a stable layer of gold. However, gold surfaces face issues like formation of reactive oxygen species on gold at negative voltages,^19^ susceptibility to biofouling,^41,42^ and parasitic nonspecific reactions in complex matrices.^43^ To overcome these inherent limitations of gold surfaces, we explored the use of highly porous gold (hPG), which offers a unique combination of properties like resistance to biofouling^42,44,45^ and signal amplification. This dual advantage presents an attractive platform for commercialization of biosensors.^46^ The enhanced antibiofouling behavior of hPG arises from two key features: its increased electroactive surface area, which improves the signal-to-noise ratio and enables efficient analyte–surface interactions even when some fouling occurs,^47^ and its nanoscale porosity,^48,49^ which contributes to a quasi-size-exclusion effect, selectively impeding access of larger fouling species such as proteins or lipids while permitting smaller, electroactive analytes like phosphorylated tau peptides to reach the electrode surface. This porosity also modifies surface energy and hydrophobicity, further discouraging nonspecific adsorption.^50^ These factors combine to provide improved long-term electrochemical performance in complex biological matrices, such as plasma.

To translate this promising hPG/pPhR-pPy nMIP platform into a practical and effective point-of-care diagnostic tool for AD diagnosis, our development focused on creating a fully integrated and intelligent system. A cornerstone of this system is a homemade, compact potentiostat, meticulously engineered for precise electrochemical control and sensitive signal acquisition tailored for portable point-of-need applications. Complementing this, the significance of machine learning (ML) is paramount in extracting clear diagnostic insights from sensor data. Our ML approach uniquely integrates two critical functions: first, a classification model, evaluated via confusion matrix analysis, accurately determines the presence or absence of the p-tau181 protein on the nMIP. Second, a quantitative model, whose output can be visualized as a heat map, precisely determines the concentration of the protein captured on the surface. This sophisticated, two-part ML strategy is crucial, as it directly addresses the challenge of diagnostic accuracy by significantly reducing false positives and negatives, thereby achieving substantially better and more reliable sensing performance compared to conventional analytical methods.

The synergy of molecularly imprinted polymers (MIPs) with ML is increasingly recognized for creating robust biosensors that tackle complexities like matrix effects and interference in complex samples.^51^ Wang et al. developed a voltammetric electronic tongue employing electropolymerized pPy-MIPs on Au nanoparticle-decorated multiwall carbon nanotubes (Au-fMWCNTs), coupled with artificial neural networks, for the simultaneous analysis of three fluoroquinolone antibiotics. While innovative in its array format and ML application for resolving mixtures, its electrochemical characterization steps involved external redox probes like ferricyanide to follow the sensor modification process.^52^ More recently, Dashtaki et al. focused significantly on enhancing the predictive performance and reliability of a pPy MIP sensor for doxorubicin detection by developing a sophisticated stacking regressor ensemble of multiple ML models and employing feature engineering techniques such as SHAP analysis.^53^ These studies underscore the power of ML in advanced sensor systems. However, a distinct need persists for fully integrated, label-free point-of-care (PoC) platforms where novel, intrinsically redox-active MIPs are combined with ML algorithms specifically architected for direct diagnostic decision-making, particularly for challenging, low-concentration clinical biomarkers.

Bridging the gap from these foundational lab-scale advancements to tangible clinical tools, our study distinctively advances the application of MIPs and AI by presenting a fully integrated, scalable, and low-cost PoC biosensor specifically engineered for the early detection of AD through the critical biomarker phosphorylated Tau 181 (p-tau181). Unlike many systems explored at the lab scale, our platform features a novel nMIP transducer with intrinsic redox activity, fabricated on a cost-effective PCB with enhanced hPG electrodes. This sensor demonstrates exceptional performance not only in undiluted plasma and serum but, significantly, in clinical samples from AD patients. Coupled with a custom-engineered compact potentiostat and a sophisticated dual-function ML algorithm (for robust classification and precise quantification), our system achieves a detection limit of 498 fg mL^–1^, a high sensitivity of 8.987 μA/pg mL^–1^, and a diagnostic receiver operating characteristic area under the curve (ROC AUC) of 0.84. This remarkable performance, validated against and proving comparable to gold-standard single-molecule array (SIMOA), is delivered at a fraction of the cost. These results firmly establish our platform as a significant leap from prior MIP-ML explorations toward truly accessible, rapid, and affordable point-of-care Alzheimer’s diagnostics, with strong potential for widespread implementation. While prior work has explored MIPs and ML separately, our fully integrated system demonstrates real-world clinical validation for a phosphorylated protein biomarker at femtomolar levels in complex matrices.

## Experimental Section

### Materials

Silver and gold brush plating solutions were supplied by Spa Plating (Bath, UK). Gold­(III) chloride, ammonium chloride, phenol red sodium salt (selected for its redox activity and compatibility with polymerization techniques), ferric chloride, and oxalic acid were sourced from Sigma-Aldrich (St. Louis, MO, USA). Recombinant p-tau181 protein was purchased from Abcam (Cambridge, UK). Printed circuit boards (PCBs) with ENIG surface finish was obtained from JLC PCB (Shenzhen, China).

### Electrode Preparation

The PCB electrode design consisted of two working electrodes (WE1 and WE2) of size 3 mm × 1.5 mm, one counter electrode (CE) of size 7 mm × 2 mm island, and one reference electrode (RE) of size 2 mm × 1 mm island. A silver layer was deposited on all electrodes using a silver brush plating solution (SPA plating) to improve surface planarity and ensure signal reproducibility. Silver also acts as a barrier layer to the ENIG surface as the nickel in this composite is electrochemically active and renders the surface unsuitable for performing electrochemical measurements. A potentiostatic cell was setup with the potentiostat (Palmsense Emstat 4) WE connected to the PCB islands, RE connected to a standard glass junction Ag/AgCl reference electrode, and the CE was connected to a Pt/Ti-type rod. Vigorous stirring was achieved by using a magnetic stirrer set at 300 rpm. Multistep amperometry was performed at −0.5 and −1.2 V in 2.5 s pulses for 16 cycles to achieve uniform silver deposition.

For gold electroplating, chronopotentiometry was performed at −1.5 mA for 300 s using a gold brush plating solution (Spa Plating) in a galvanic cell with a Pt/Ti CE. The RE island was chloridized by immersion in 100 mM ferric chloride solution for 30 s, forming a stable silver chloride layer.

### Highly Porous Gold (hPG) Surface Modification

To further optimize the sensing surface, a hPG layer was electrodeposited onto the WE. A potentiostatic setup was employed, with a Pt counter electrode (CE) and a standard glass junction Ag/AgCl RE.

Soft gold was deposited by reducing gold­(III) chloride, using cyclic voltammetry (CV) between 0.8 and 0.3 V at a scan rate of 0.05 V/s for 10 cycles in a freshly prepared 10 mM gold­(III) chloride and 2.5 M ammonium chloride solution (1:1 ratio). A chronoamperometric deposition step at – 0.9 V for 90 s was used to generate a hPG layer by hydrogen bubble templating.

### Deposition of the Nanomolecularly Imprinted Polymer (nMIP)

To fabricate the pPhR-pPy composite, a 1 mM pPhR solution and 8 mM Py solution were prepared in 10 mM phosphate-buffered saline (PBS) and degassed under nitrogen for 15 min. Recombinant p-tau181 protein (0.2 mg mL^–1^, 5 μL) was mixed with 200 μL of pPhR solution and 795 μL of PBS, resulting in a final concentration of 1 μg mL^–1^.

Electropolymerization was performed using a PCB-based WE, a platinum wire CE, and a Ag/AgCl RE. The CV for the electropolymerization was applied from 0.3 to 0.9 V at a scan rate of 0.1 V/s for 18 cycles. The imprinted electrodes were then incubated overnight in 50 mM oxalic acid at 3.5 °C to remove the template molecule, leaving high-affinity binding cavities for p-tau181.

The schematic illustrates the sequential construction of the molecularly imprinted polymer (MIP)-based electrochemical sensor platform along with the interaction mechanism between phenol red (PhR) and pyrrole (Py) and between PhR/Py and the target protein (Figure 1). Hydrogen bonds are indicated in blue dashes, and π–π interactions are indicated in red dashes. Starting from bare Au electrodes, a highly porous gold (hPG) layer is deposited via electrodeposition and electroporation to increase the electroactive surface area. This is followed by electropolymerization of pPhR and pPy in the presence (for MIP) or absence (for NIP) of the target template protein (p-tau181) to form the sensing layer. The final MIP@hPG sensor is obtained after oxalic acid treatment, which removed the p-tau181 template to leave selective molecular recognition cavities. This hierarchical architecture—comprising hPG for enhanced sensitivity and PhR/pPy MIP for target specificity—enables ultrasensitive, selective detection of Alzheimer’s biomarkers in clinical samples.

**Figure 1:**
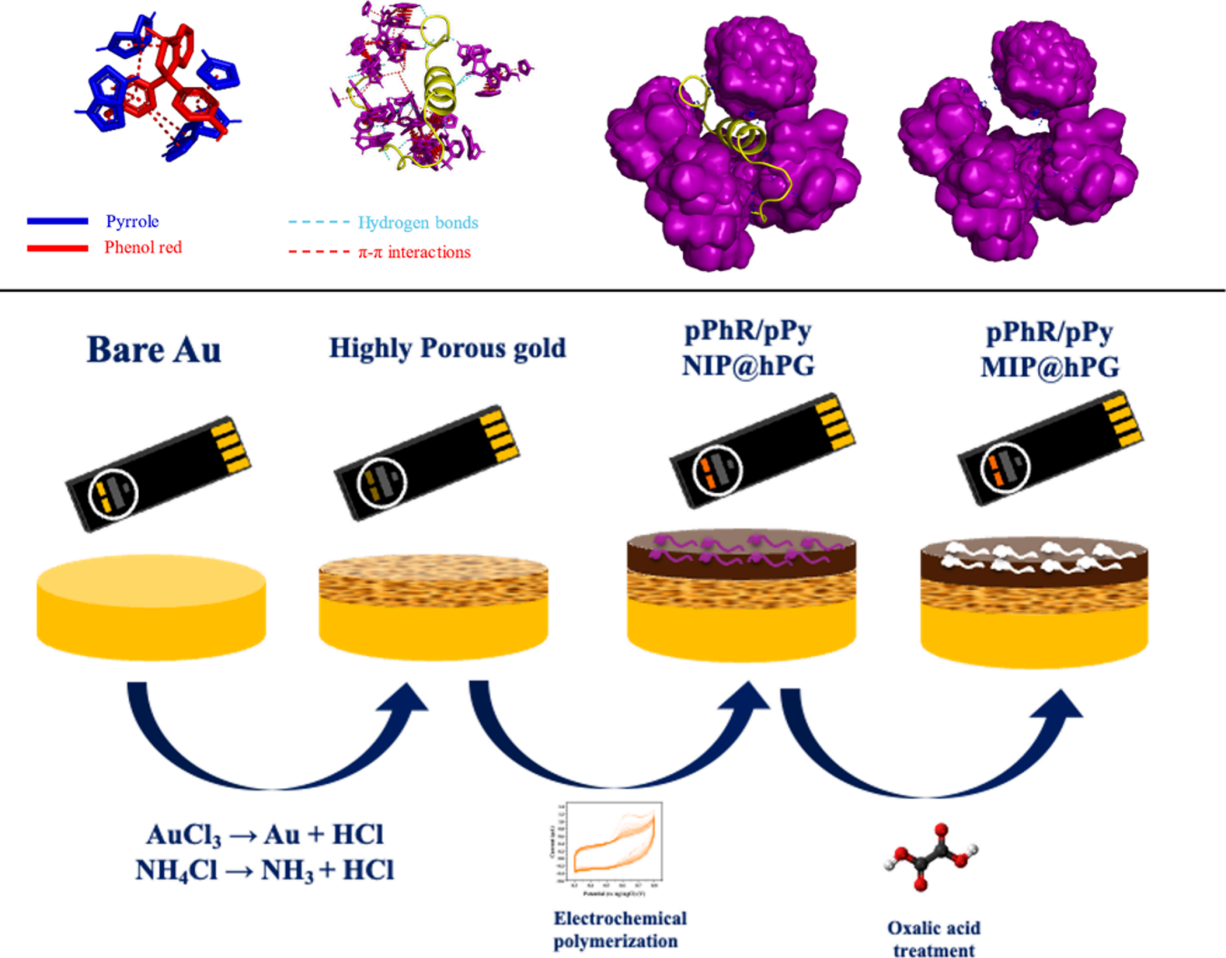
Stepwise fabrication of the redox-active, highly porous gold-based MIP biosensor for p-tau181 detection.

### Characterization Techniques

#### Scanning Electron Microscopy (SEM) and Energy-Dispersive X-ray Spectroscopy (EDS)

The structural and elemental composition of the sensor surface was characterized by using a ZEISS Gemini 2 SEM/EDS system. To enhance imaging resolution and reduce drift under high vacuum, samples were sputter-coated with chromium before analysis.

#### Fourier Transform Infrared Spectroscopy (FTIR) and Raman Spectroscopy

FTIR spectra were recorded using a Bruker Vertex 70 spectrometer equipped with a Diamond MIRacle ATR accessory (Pike Technologies) and a deuterated l-alanine-doped triglycine sulfate (DLaTGS) detector. Spectra were collected over 4000–400 cm^–1^ with a 4 cm^–1^ resolution, averaging 32 scans per spectrum. Data processing was performed by using Bruker OPUS 8.2 software.

Raman spectroscopy was conducted using a Renishaw InVia Raman microscope with a 532 nm laser (10% power, 1 s exposure, and measurement range 1570–350 cm^–1^). Raman mapping was performed in StreamHR mode using a 10× objective, with the instrument calibrated to a silicon standard (520.5 cm^–1^). The spatial distribution of polymer components was analyzed using Renishaw’s empty modeling method.

#### Electrochemical Measurements and Studies

Electrochemical studies were conducted using a Palmsense EmStat4 potentiostat, measuring the coating, poration, and deposition of the sensor components.

Differential pulse voltammetry (DPV) and CV were used to assess the sensor performance in undiluted human plasma spiked with p-tau181. DPV measurements were performed from 0.1 to 0.5 V, while CVs were recorded from −0.2 to 0.4 V at a scan rate of 0.1 V/s.

To evaluate sensor reproducibility, 400 independent biosensors were fabricated and their electrochemical response was analyzed using ferricyanide in 0.1 M PBS. The variation in Δ*I* and Δ*E* across the sensors was analyzed using probability distribution studies, ensuring batch-to-batch consistency.

Additionally, to assess the sensor’s stability during transportation, DPV measurements were recorded before and after air transport to Japan, with changes in current response analyzed as a function of % signal deviation.

### Bioinstrumentation

#### Wireless Handheld Potentiostat and Multiplexer

A custom-designed handheld potentiostat was developed to interface with the nanoMIP-modified electrodes. The functional block diagram of the device is shown in Figure S5. The system architecture integrates an analogue front end (AFE), a power management system (PMS), and a microcontroller unit (ESP32). The AFE was built around the LM324N quad operational amplifier for cost-effective signal conditioning. Channel 1 was configured as a differential amplifier, receiving input from both the DAC-generated control potential and the buffered reference electrode signal. Channel 2 acted as a high-impedance buffer to preserve the RE signal integrity. Channel 3 was configured as a transimpedance amplifier (TIA) for converting the working electrode current to voltage, subsequently digitized by a 12-bit ADC. Key operating equations include the following:

Differential amplifier

$$V_{\mathrm{CE}}=-V_{\mathrm{inv}}+(V_{\mathrm{ref}}+V_{\mathrm{RE}})$$

Transimpedance amplifier

$$V_{\mathrm{TIA}}=I_{\mathrm{WE}}\times R_{f}$$

The PMS used a TEA-10505 DC–DC isolator to generate ±5 V rails and analogue ground, enabling dual-supply operation for the AFE. The microcontroller included an integrated real-time clock (RTC) for precise signal timing and Wi-Fi capabilities for data transmission. The digitized output was parsed into a JSON object containing potential–current pairs, which were then analyzed through the ML pipeline via the custom web app.

A mechanical multiplexer relay controlled via GPIO pins enabled sequential acquisition from two working electrodes (WE1 and WE2). The default connection was to WE1 (NC), switching to WE2 (NO) upon the application of a high logic signal.

The handheld potentiostat was validated using cyclic voltammetry (CV) in a standard 5 mM [Fe­(CN)_6_]^3–^/^4–^ and 0.1 M KCl system on bare gold electrodes (−0.1 to 0.5 V vs Ag/AgCl) and benchmarked against two commercial systems (CHI030a and PalmSens EmStat 4). Consistent redox peak profiles across devices confirm signal fidelity. Dose–response studies in p-tau181-spiked plasma were also performed using the handheld system after transport to verify robustness under field conditions.

#### Machine Learning Web App

A supervised ML pipeline was implemented using DecisionTree and RandomForest classifiers from scikit-learn, enabling predictive modeling and false positive mitigation. Feature extraction algorithms generated feature vectors, which were trained and deployed within a Flask-based web application served by Gunicorn (WSGI).

The system ingested JSON data from the potentiostat, performed automated classification, and presented results via a web-based dashboard, offering a real-time analysis of p-tau181 levels.

#### Quantification of p-tau181 with a Conventional Immunoassay in Human Plasma Obtained from Patients with AD and Controls

Pilot plasma samples of patients with AD (*n* = 10, mean age [range]: 70.1 [67–72], 5 males and 5 females) and age-matched normal controls (*n* = 10, mean age [range]: 70.1 [67–72], 5 males and 5 females) were purchased from KAC Co., Ltd. (Tokyo, Japan). Informed consent was obtained by KAC Co., Ltd. from blood transfusion donors for the use of excess blood samples for research purposes. AD patients were clinically diagnosed by experienced doctors. The plasma samples were collected in EDTA-containing tubes, stored in polypropylene vials at–80 °C, and then transferred to the National Institute for Quantum Science and Technology (QST, Chiba, Japan). Plasma p-tau181 levels were measured using a SIMOA p-tau181 Advantage Kit v2.1 (Quanterix) on a SIMOA HD-X analyzer (Quanterix) at the Department of Functional Brain Imaging, QST. On the day of the analysis, the samples were thawed at room temperature, vortexed at 2000 rpm for 30 s, and then centrifuged at 4000*g* for 10 min. Thereafter, four dilutions of the plasma samples were prepared with the sample diluent before the analysis. All plasma samples were analyzed on the same day using the same number of reagents for the assay to avoid assay variability caused by lot differences.

## Results and Discussion

### Surface Morphology and Chemical Characterization

The morphology of the sensor surface was studied by using a scanning electron microscope (SEM). The gold-coated PCB electrode (Figure 2A) exhibited a smooth surface with low roughness (∼3–4 nm). Upon modification with hPG (Figure 2B), the surface roughness increased to approximately 10 nm, indicating successful nanostructuring. The deposition of pPhR onto hPG (Figure 2C) resulted in distinct nanoscale features (∼15–20 nm in size). The incorporation of pPy into the composite (pPhR-pPy) (Figure 2D) further increased feature size to 25–30 nm, suggesting successful polymer integration and enhanced surface conductivity.^54,55^

**Figure 2:**
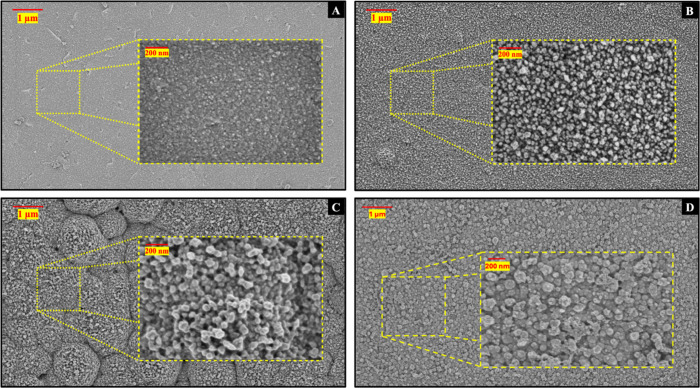
SEM images of (A) Au coated on the PCB electrode, (B) hPG, (C) pPhR coated on hPG, and (D) pPhR-pPy coated on hPG.

To investigate chemical interactions within the pPhR-pPy composite, Fourier transform infrared spectroscopy (FTIR) was conducted (Figure 3A). Peaks at 1168–1195 cm^–1^ correspond to sulfonate groups from pPhR, while additional bands characteristic of C=C stretching, C–N bonds, and N–H vibrations confirm pyrrole incorporation (Figure 3A). Bands in the range of 1168–1195 cm^–1^ can be attributed to the sulfonate groups present in the pPhR (red line) and pPhR-pPy (black line) composite.^30^ Additionally, FTIR also shows peaks characteristic of the C=C stretching, =C–H in-plane vibration, =C–H out-of-plane vibrations, C–N stretch, N–H in-plane vibrations, and ring deformations, all of which correspond to the presence of pyrrole in the composite.^56–59^ There are slight shifts in the peak when polymerization is carried out in the absence of pPy, which implies the functionality of pPy in the composite.

**Figure 3:**
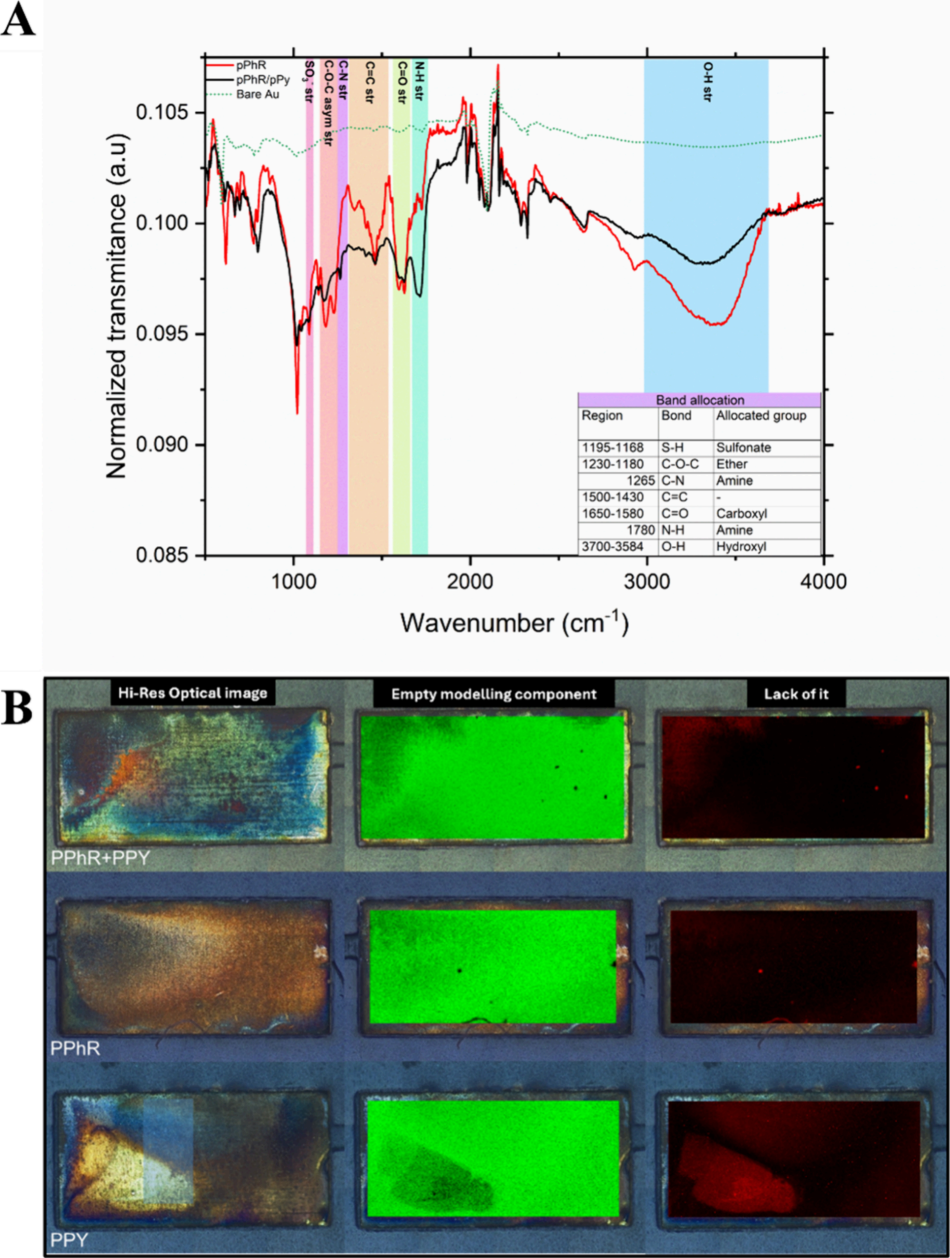
(A) FTIR spectra of the pPhR-pPy (black line) composite on the hPGAu substrate in comparison to individual constituents (pPhR (red line) and bare Au (green dotted line)); (B) image of Raman mapping using a Renishaw empty model, depicting the optical image and the uniformity of the polymer.

Raman spectroscopy was used to assess the polymer distribution on the sensing surface (Figure 3B). Raman mapping revealed a uniform distribution of pPhR-pPy, with only minimal uncoated regions likely due to surface irregularities. Energy-dispersive X-ray spectroscopy (EDS) further confirmed the presence of sulfur (S) and nitrogen (N), supporting successful functionalization (Figure S1).

The increased surface roughness observed in SEM is not directly related to intrinsic material conductivity but rather contributes to a larger electroactive surface area. This enhancement increases the number of accessible redox sites, thereby facilitating higher current densities during detection. The improved electrochemical performance is therefore attributed to geometric surface amplification rather than any change in the intrinsic conductivity of the material.

### Electrochemical Performance and Sensitivity

To evaluate the sensor performance, DPV was performed in PBS (Figure S2A) and unprocessed human plasma spiked with p-tau181 (0–22.5 pgmL^–1^) (Figure 4A). Calibration plots (Figure 4B) demonstrated that the hPG/pPhR-pPy sensor exhibited a higher signal response compared with the hPG/pPhR sensor, which can be attributed to the synergistic interplay between pPy and pPhR within the composite. The incorporation of pPy enhances conductivity through its conjugated π-electron system, which facilitates efficient electron delocalization and charge transfer between the redox-active pPhR and the porous gold electrode. This enhanced conductivity amplifies the Faradaic current generated during p-tau181 binding, directly contributing to the higher observed sensitivity (8.99 μA/pg mL^–1^), a substantial improvement over the hPG/pPhR sensor (4.39 μA/pg mL^–1^). The dynamic range (1–16 mL^–1^) (*R* ^2^= 0.982) aligns well with physiological p-tau181 concentrations, and a limit of detection (LOD) of 498 fg mL^–1^ was achieved, further highlighting the sensor’s superior electrochemical properties. This was calculated using the slope (*S* = 8.04) and the standard deviation of the blank (σ = 1.214 μA, *n* = 18), with the formula:

$$\mathrm{LOD}=3.3\frac{Sy}{S}=3.3\frac{1.214}{8.04}=0.498\;\mathrm{pg}\;{\mathrm{mL}}^{-1}$$

**Figure 4:**
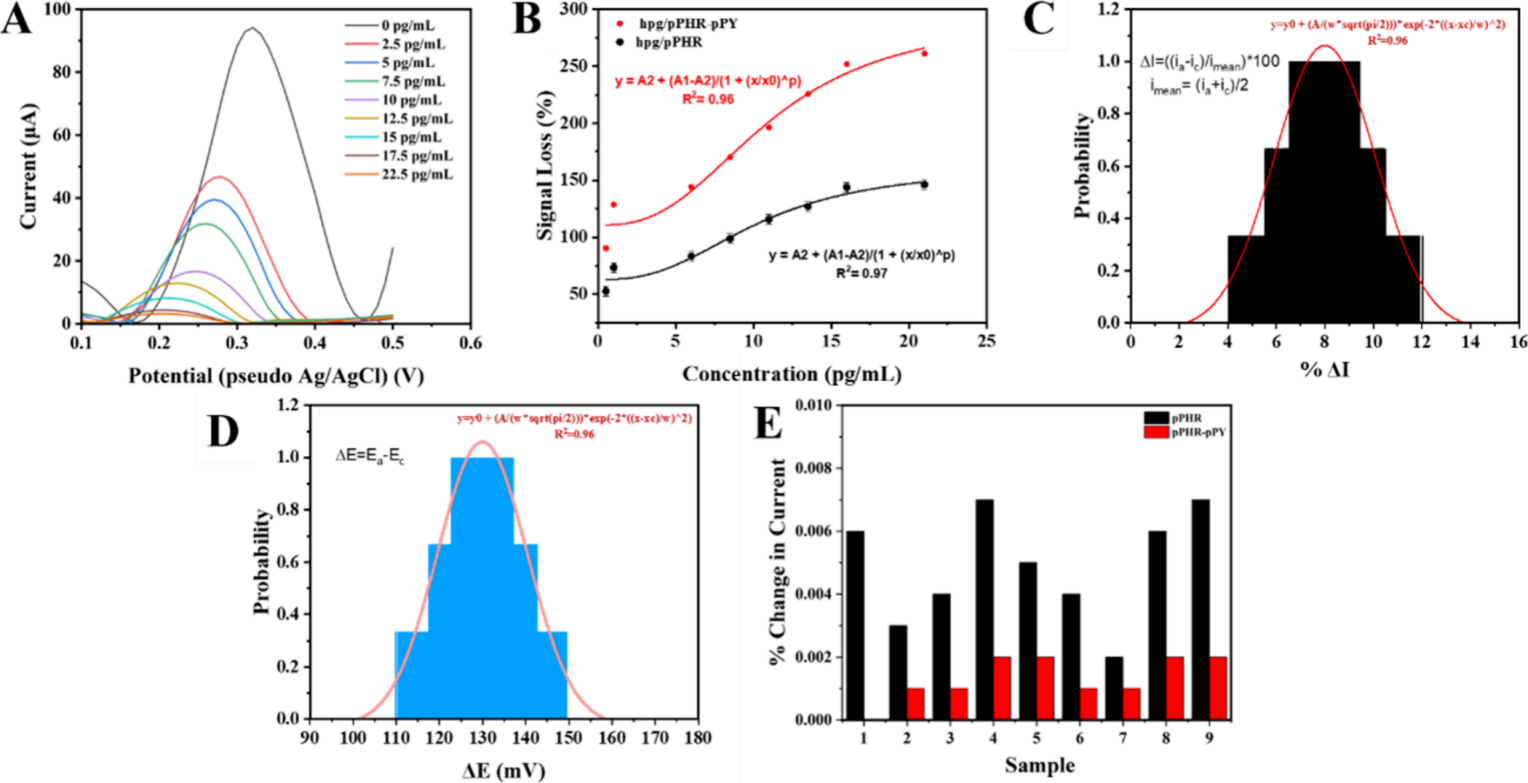
(A) DPVs of the hPG/pPhR-pPy in spiked plasma. (B) Calibration plot (in triplicate) obtained using the hPG/pPhR-pPy and hPG/pPhR in plasma containing different concentrations of p-tau-18, probability distribution of (C) Δ*I* and (D) Δ*E* among the 400 sensors, and (E) air travel stability of the pPhR-pPy nMIP as a function of % change in current before and after air travel.

Beyond electrical performance, the electropolymerized pPy framework also enhances the sensor’s structural properties. As observed in the SEM images (Figure 2D), the nanostructured pPhR-pPy composite increases the effective surface area, thereby boosting the density of imprinted cavities available for target capture. Additionally, pPy imparts mechanical robustness, stabilizing the composite and mitigating pPhR’s inherent susceptibility to environmental degradation, such as pH fluctuations and thermal stress. To assess sensor reproducibility, 400 independent biosensors were fabricated and analyzed using cyclic CV in a ferricyanide solution (Figure S2B). The probability distributions of Δ*I* and Δ*E* (Figure 4C,D) followed by a Gaussian profile, indicating high reproducibility across multiple batches.

A crucial factor for real-world applicability is the sensor stability during transportation. To evaluate this, DPV measurements were taken before and after the international air transport to Japan. The hPG/pPhR-pPy sensor exhibited greater stability compared to the hPG/pPhR sensor, as evidenced by a lower percentage signal deviation (Figure 4E). This improved transport resilience is likely due to the ability of the pPy to reinforce the composite matrix, preventing structural deterioration during handling and transit. Furthermore, the pPy backbone may reduce nonspecific binding via hydrophobic interactions, while the sulfonate (−SO_3_^–^) groups on pPhR provide electrostatic specificity for phosphorylated residues on p-tau181, collectively enhancing both sensitivity and selectivity. These synergistic effects are further reflected in the receiver operating characteristic (ROC) analysis, where the hPG/pPhR-pPy sensor achieved an AUC of 0.84, demonstrating a marked improvement over that of pPhR alone.

### Data Analytics and Machine Learning Performance

A supervised ML model was implemented to enhance the biosensor classification accuracy. A confusion matrix (Figure 5A) demonstrated that the model successfully identified 9 true negatives and 71 true positives out of 80 samples, indicating a high detection accuracy for p-tau181. The test–train split analysis (Figure 5B) revealed a consistent predictive performance, with some variance observed at higher p-tau181 concentrations (13.5–21 pg mL^–1^).

**Figure 5:**
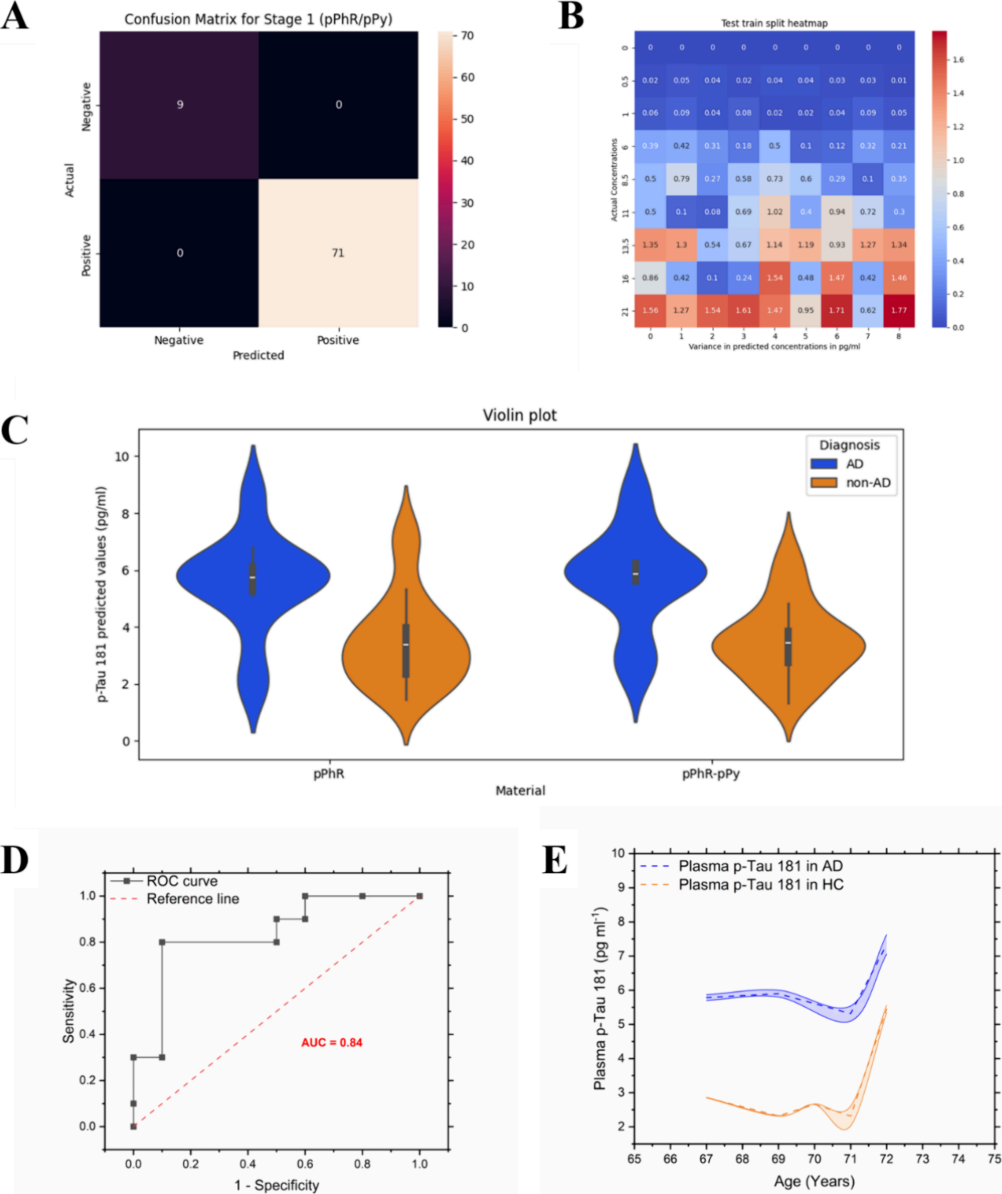
(A) Confusion matrix output used to cross-validate the classifier, (B) heat map displaying variance of data vs expected values for the regressor in the case of hPG/pPhR-pPy, (C) violin plot, (D) ROC plots for the hPG/pPhR-pPy, and (E) time plots of plasma protein levels (pg mL^–1^) showing the 5 year rolling means for control/non-AD (yellow) and case (blue) groups with 90% confidence intervals on the mean shown (shaded areas).

To compare the distinction capability of hPG/pPhR and hPG/pPhR-pPy sensors, we employed violin plots (Figure 5C). These plots highlight an increased separation and reduced overlap in the hPG/pPhR-pPy data set, confirming superior diagnostic accuracy. The receiver operating characteristic (ROC) curve (Figure 5D) yielded an area under the curve (AUC) of 0.84 in comparison to 0.81 exhibited by the pPHR sensor (Figure S3A), demonstrating a strong classification ability. A direct comparison with SIMOA (AUC 0.91, Figure S3B) showed comparable performance, positioning the pPhR-pPy biosensor as a viable, low-cost alternative.

Further, age-resolved plasma p-tau181 levels (Figure 5E) indicated that AD cases show notable biomarker elevation from around 71 years of age, with an estimated 26% increase in p-tau181 levels at age 72. This observation is based on a limited data set and warrants further study in larger age-stratified cohorts. The findings suggest that this biosensor could be implemented as a triaging tool for AD detection, providing an accessible alternative to high-cost laboratory assays.

To ensure statistical rigor and model transparency, we evaluated both the regression and classification performance using standard machine learning metrics. For the classification model, the test data set yielded an *F*1 score of 1.00, an accuracy of 1.00, a precision of 1.00, and a recall of 1.00, indicating excellent discrimination between p-tau181-positive and control samples. For the regression model, we observed a mean absolute error (MAE) of 0.35 pg mL^–1^, a mean squared error (MSE) of 0.66, a root mean squared error (RMSE) of 0.81, and a coefficient of determination (*R* ^2^) of 0.96. Feature extraction was performed using a chemometric algorithm that parsed differential pulse voltammetry (DPV) curves into peak-related descriptors. A total of 2750 electrochemical data points from spiked plasma and serum were used to train the model. The data set was split into a 75:25 train–test ratio using stratified sampling via scikit-learn’s train_test_split function. Both models were also validated using five-fold cross-validation to confirm robustness. A summary of these performance metrics is provided in Tables S1 and S2 (Supporting Information).

### Comparative Analysis with SIMOA and Clinical Relevance

While SIMOA remains the gold-standard immunoassay for p-tau181 detection, its high cost (∼£6000 per 78-well plate), labor-intensive workflow, and requirement for specialized operators pose significant barriers to widespread clinical adoption. In contrast, our hPG/pPhR-pPy sensor presents a cost-effective and scalable alternative, offering the following:

- comparable diagnostic performance (AUC 0.84 vs SIMOA 0.91),
- significantly lower cost (∼£20 per test),
- point-of-care usability with no need for trained personnel, and
- rapid detection (<10 min per test).

These advantages position the hPG/pPhR-pPy biosensor as a viable, field-deployable diagnostic tool, particularly for resource-limited healthcare settings where access to conventional, high-cost assays is restricted. Future studies will focus on validating the sensor’s performance in larger clinical cohorts, refining AI-driven analytics, and integrating the technology into portable diagnostic platforms to enhance accessibility and real-world applicability.

While the sensor demonstrated strong performance in undiluted plasma, comprehensive cross-reactivity testing with structurally related proteins and abundant plasma constituents remains an important future step. In this study, the limited availability of recombinant proteins and clinical sample volume constrained such analyses. However, a preliminary comparison using Tau441 and p-tau181 recombinant proteins revealed that the pPhR-pPy MIP sensor exhibited a markedly higher response to p-tau181, suggesting favorable molecular recognition (Figure S4). Nevertheless, we acknowledge that further work is required to systematically evaluate potential interference from proteins such as albumin, transferrin, and additional phosphorylated or nonphosphorylated tau isoforms. These selectivity profiling studies are planned for future investigations to fully characterize the specificity and diagnostic reliability of the sensors in complex clinical matrices.

## Conclusions

This study presents an nMIP-based electrochemical biosensor for the detection of phosphorylated Tau 181 (p-tau181) in undiluted plasma, offering a low-cost, highly sensitive alternative to conventional immunoassays. The pPhR-pPy composite demonstrated superior electrochemical properties, achieving a sensitivity of 8.987 μA/pg mL^–1^ and an LOD of 498 fg mL^–1^, making it comparable to SIMOA yet significantly more cost-effective (under £20 per test versus ∼£6000 per 78-well plate). The LOD was calculated using the 3.3 Sy criterion, where σ is the standard deviation of the blank signal and the slope was obtained from the linear portion of the calibration curve.

The enhanced performance of the pPhR-pPy sensor over pPhR alone arises from the synergistic interplay of its constituent polymers. The pPy facilitates electron transfer kinetics through its conductive backbone while also providing mechanical stability and a nanostructured morphology, increasing the surface area and accessibility of binding sites. The pPhR contributes to redox activity and electrostatic specificity, enabling label-free detection of p-tau181. The integration of pPy mitigates the inherent limits of pPhR such as nonconductivity and environmental instability, resulting in a sensor with higher sensitivity, lower LOD, and robust reproducibility across fabrication batches. This composite design exemplifies a strategic approach to optimizing synthetic receptors for point-of-care diagnostics, balancing electrochemical performance with practical durability.

By integration of an hPG electrode, a custom-designed potentiostat, and ML-driven data analytics, this biosensor overcomes biofouling, signal instability, and scalability challenges common to electrochemical detection systems. The ROC curve analysis yielded an AUC of 0.84, indicating a strong diagnostic potential, particularly for PoC applications. Furthermore, age-resolved data suggest a significant rise in p-tau181 levels around 71 years of age, reinforcing the sensor’s potential for early-stage AD triaging.

This work establishes a scalable, rapid, and accessible AD diagnostic platform, bridging the gap between high-performance laboratory assays and real-world clinical deployment. Future research should focus on clinical validation with larger cohorts, further optimization of AI-driven data processing, and integration into wearable or portable devices to expand the global accessibility of AD diagnostics.

## Supplementary Material



## Notes

Additional experimental data; (Figures S1–S7) EDS spectra of the polyphenol–polypyrrole composite, differential pulse voltammetry and cyclic voltammetry response curves, receiver operating characteristic (ROC) curves, and handheld device design and validation results; (Tables S1–S4) classification and regression model performance metrics and intra- and interassay variance data (PDF)

## References

[1] Nichols E., Szoeke C. E. I., Vollset S. E., Abbasi N., Abd-Allah F., Abdela J., Aichour M. T. E., Akinyemi R. O., Alahdab F., Asgedom S. W. (2019). Global, regional, and national burden of Alzheimer’s
disease and other dementias, 1990–2016: a systematic analysis
for the Global Burden of Disease Study 2016. Lancet Neurology.

[2] Lanctôt K. L., Hahn-Pedersen J. H., Eichinger C. S., Freeman C., Clark A., Tarazona L. R. S., Cummings J. (2024). Burden of
Illness in People with
Alzheimer’s Disease: A Systematic Review of Epidemiology, Comorbidities
and Mortality. J. Prev. Alzheimer’s Dis..

[3] Edwards S., Trepel D., Ritchie C., Hahn-Pedersen J. H., Robinson D. E., Chan M. S., Bray B. D., Clark A., Ivkovic M., Michalak W. (2024). Real world
outcomes,
healthcare utilisation and costs of Alzheimer’s disease in
England. Aging and Health Research.

[4] Nazir S. (2024). Salivary biomarkers:
The early diagnosis of Alzheimer’s disease. AGING MEDICINE.

[5] Ashton N. J., Brum W. S., Di Molfetta G., Benedet A. L., Arslan B., Jonaitis E., Langhough R. E., Cody K., Wilson R., Carlsson C. M. (2024). Diagnostic
Accuracy of a Plasma Phosphorylated
Tau 217 Immunoassay for Alzheimer Disease Pathology. JAMA Neurology.

[6] Frisoni G. B., Festari C., Massa F., Cotta Ramusino M., Orini S., Aarsland D., Agosta F., Babiloni C., Borroni B., Cappa S. F. (2024). European
intersocietal
recommendations for the biomarker-based diagnosis of neurocognitive
disorders. Lancet Neurology.

[7] Ashton N. J., Zetterberg H. (2025). A blood test
for Alzheimer’s disease: a
decade of progress and success. eBioMedicine.

[8] Abdullah S. A., Najm L., Ladouceur L., Ebrahimi F., Shakeri A., Al-Jabouri N., Didar T. F., Dellinger K. (2023). Functional
Nanomaterials for the Diagnosis of Alzheimer’s Disease: Recent
Progress and Future Perspectives. Adv. Funct.
Mater..

[9] Schindler S. E., Galasko D., Pereira A. C., Rabinovici G. D., Salloway S., Suárez-Calvet M., Khachaturian A. S., Mielke M. M., Udeh-Momoh C., Weiss J. (2024). Acceptable
performance of blood biomarker tests of amyloid pathology —
recommendations from the Global CEO Initiative on Alzheimer’s
Disease. Nature Reviews Neurology.

[10] Han Q., Wang H., Wang J. (2024). Multi-Mode/Signal
Biosensors: Electrochemical
Integrated Sensing Techniques. Adv. Funct. Mater..

[11] Zu L., Wang X., Liu P., Xie J., Zhang X., Liu W., Li Z., Zhang S., Li K., Giannetti A. (2024). Ultrasensitive and Multiple Biomarker
Discrimination for Alzheimer’s
Disease via Plasmonic & Microfluidic Sensing Technologies. Advanced Science.

[12] Rezabakhsh A., Rahbarghazi R., Fathi F. (2020). Surface plasmon resonance biosensors
for detection of Alzheimer’s biomarkers; an effective step
in early and accurate diagnosis. Biosens. Bioelectron..

[13] Wang S., Zhang R., Li X., Chen Y., Zhu L., Yang B., Wang J., Du Y. h., Liu J., Ye T. t. (2024). “Rigid-Flexible”
Dual-Ferrocene Chimeric
Nanonetwork for Simultaneous Tumor-Targeted Tracing and Photothermal/Photodynamic
Therapy. ACS Appl. Mater. Interfaces.

[14] Jin Z., Yim W., Retout M., Housel E., Zhong W., Zhou J., Strano M. S., Jokerst J. V. (2024). Colorimetric sensing for translational
applications: from colorants to mechanisms. Chem. Soc. Rev..

[15] Li Y., Luo L., Kong Y., Li Y., Wang Q., Wang M., Li Y., Davenport A., Li B. (2024). Recent advances in molecularly imprinted
polymer-based electrochemical sensors. Biosens.
Bioelectron..

[16] Tong J., Li C., Zhao J., Wang K., Zhao Z., Liu Y., Qing T., Liu X. (2024). Poly-Adenine Assisted Signaling Displaced
Probe Ratiometric Electrochemical Aptasensor for Accurate Detection
of Alzheimer’s Disease Aβ Biomarkers. ACS Appl. Mater. Interfaces.

[17] Sun Z., Zhang B., Tu H., Pan C., Chai Y., Chen W. (2024). Advances in colorimetric biosensors
of exosomes: novel approaches
based on natural enzymes and nanozymes. Nanoscale.

[18] Chen X., Huang Y., Yang S., Wang S., Chen L., Yu X., Gan N., Huang S. (2024). In-situ nanozyme catalytic amplification
coupled with a universal antibody orientation strategy based electrochemical
immunosensor for AD-related biomarker. Biosens.
Bioelectron..

[19] Arroyo-Currás N. (2024). Beyond the
Gold–Thiol Paradigm: Exploring Alternative Interfaces for Electrochemical
Nucleic Acid-Based Sensing. ACS Sensors.

[20] Alanazi K., Garcia Cruz A., Di Masi S., Voorhaar A., Ahmad O. S., Cowen T., Piletska E., Langford N., Coats T. J., Sims M. R. (2021). Disposable paracetamol sensor based on electroactive
molecularly imprinted polymer nanoparticles for plasma monitoring. Sensors Actuators B: Chem..

[21] Wang M., Ye C., Yang Y., Mukasa D., Wang C., Xu C., Min J., Solomon S. A., Tu J., Shen G. (2025). Printable
molecule-selective core–shell nanoparticles for wearable and
implantable sensing. Nat. Mater..

[22] Li Y., Guan C., Liu C., Li Z., Han G. (2024). Disease diagnosis
and application analysis of molecularly imprinted polymers (MIPs)
in saliva detection. Talanta.

[23] Haq I., Alanazi K., Czulak J., Di Masi S., Piletska E., Mujahid A., Hussain T., Piletsky S. A., Garcia-Cruz A. (2021). Determination
of sitagliptin in human plasma using a smart electrochemical sensor
based on electroactive molecularly imprinted nanoparticles. Nanoscale Advances.

[24] Li P., Liu Z. (2024). Glycan-specific molecularly
imprinted polymers towards cancer diagnostics:
merits, applications, and future perspectives. Chem. Soc. Rev..

[25] Arjun A. M., Deshpande S., Dunlop T., Norman B., Oliviera D., Vulpe G., Moreira F., Sharma S. (2024). Alzheimer’s
diagnosis beyond cerebrospinal fluid: Probe-Free Detection of Tau
Proteins using MXene based redox systems and molecularly imprinted
polymers. Biosensors and Bioelectronics: X.

[26] Goyal A., Sakata T. (2022). Development of a Redox-Label-Doped
Molecularly Imprinted
Polymer on β-Cyclodextrin/Reduced Graphene Oxide for Electrochemical
Detection of a Stress Biomarker. ACS Omega.

[27] Pereira M. V., Marques A. C., Oliveira D., Martins R., Moreira F. T. C., Sales M. G. F., Fortunato E. (2020). Paper-Based Platform with an In Situ
Molecularly Imprinted Polymer for β-Amyloid. ACS Omega.

[28] Lamaoui A., Palacios-Santander J. M., Amine A., Cubillana-Aguilera L. (2021). Molecularly
imprinted polymers based on polydopamine: Assessment of non-specific
adsorption. Microchem. J..

[29] Li K., Xu F., Eriksson K. E. L. (1999). Comparison
of Fungal Laccases and
Redox Mediators in Oxidation of a Nonphenolic Lignin Model Compound. Appl. Environ. Microbiol..

[30] Malarat N., Soleh A., Saisahas K., Samoson K., Promsuwan K., Saichanapan J., Wangchuk S., Meng L., Limbut W. (2024). Electropolymerization
of poly­(phenol red) on laser-induced graphene electrode enhanced adsorption
of zinc for electrochemical detection. Talanta.

[31] Chauhan R., Gill A. A. S., Nate Z., Karpoormath R. (2020). Highly selective
electrochemical detection of ciprofloxacin using reduced graphene
oxide/poly­(phenol red) modified glassy carbon electrode. J. Electroanal. Chem..

[32] Zhang M., Yang Y., Wang Y., Zhang B., Wang H., Fang G., Wang S. (2022). A molecularly
imprinted electrochemical
sensor based on cationic intercalated two-dimensional titanium carbide
nanosheets for sensitive and selective detection of triclosan in food
samples. Food Control.

[33] Unger C., Lieberzeit P. A. (2021). Molecularly
imprinted thin film surfaces in sensing:
Chances and challenges. React. Funct. Polym..

[34] Özcan S., Kaynak M. S. (2025). Structural characterization
of degradation products
of phenol red used as zero permeability marker in in-situ rat intestinal
permeability studies by LCMS-IT-TOF. J. Chromatogr.
B.

[35] Lach P., Garcia-Cruz A., Canfarotta F., Groves A., Kalecki J., Korol D., Borowicz P., Nikiforow K., Cieplak M., Kutner W. (2023). Electroactive molecularly
imprinted polymer nanoparticles for selective glyphosate determination. Biosens. Bioelectron..

[36] Ben
Hassine A., Raouafi N., Moreira F. T. C. (2023). Novel biomimetic
Prussian blue nanocubes-based biosensor for Tau-441 protein detection. J. Pharm. Biomed. Anal..

[37] Xiao X., Yan X., Magner E., Ulstrup J. (2021). Polymer coating
for improved redox-polymer-mediated
enzyme electrodes: A mini-review. Electrochem.
Commun..

[38] Zhang D., Qiu S., Huang W., Yang D., Wang H., Fang Z. (2019). Mechanically
strong and electrically stable polypyrrole paper using high molecular
weight sulfonated alkaline lignin as a dispersant and dopant. J. Colloid Interface Sci..

[39] Dianatdar A., Miola M., De Luca O., Rudolf P., Picchioni F., Bose R. K. (2022). All-dry, one-step
synthesis, doping and film formation
of conductive polypyrrole. Journal of Materials
Chemistry C.

[40] Shamkhalichenar H., Bueche C. J., Choi J.-W. (2020). Printed Circuit Board (PCB) Technology
for Electrochemical Sensors and Sensing Platforms. Biosensors.

[41] Patel J., Radhakrishnan L., Zhao B., Uppalapati B., Daniels R. C., Ward K. R., Collinson M. M. (2013). Electrochemical
Properties of Nanostructured Porous Gold Electrodes in Biofouling
Solutions. Anal. Chem..

[42] Liu Y., Arjun A. M., Webb S., Wolfe M., Chávez J. L., Swami N. S. (2024). Redox cycling-based
signal amplification at alkanethiol
modified nanoporous gold interdigitated microelectrodes. Anal. Chim. Acta.

[43] Russo M. J., Han M., Desroches P. E., Manasa C. S., Dennaoui J., Quigley A. F., Kapsa R. M. I., Moulton S. E., Guijt R. M., Greene G. W. (2021). Antifouling
Strategies for Electrochemical Biosensing: Mechanisms
and Performance toward Point of Care Based Diagnostic Applications. ACS Sensors.

[44] Othman A., Bilan H. K., Katz E., Smutok O. (2022). Highly Porous Gold
Electrodes – Preparation and Characterization. ChemElectroChem.

[45] Bollella P., Sharma S., Cass A. E. G., Tasca F., Antiochia R. (2019). Minimally
Invasive Glucose Monitoring Using a Highly Porous Gold Microneedles-Based
Biosensor: Characterization and Application in Artificial Interstitial
Fluid. Catalysts.

[46] Bollella P., Hibino Y., Kano K., Gorton L., Antiochia R. (2018). Highly Sensitive
Membraneless Fructose Biosensor Based on Fructose Dehydrogenase Immobilized
onto Aryl Thiol Modified Highly Porous Gold Electrode: Characterization
and Application in Food Samples. Anal. Chem..

[47] Yang W., Ratinac K. R., Ringer S. P., Thordarson P., Gooding J. J., Braet F. (2010). Carbon Nanomaterials in Biosensors:
Should You Use Nanotubes or Graphene?. Angew.
Chem., Int. Ed..

[48] Ahangar L. E., Mehrgardi M. A. (2012). Nanoporous
gold electrode as a platform for the construction
of an electrochemical DNA hybridization biosensor. Biosens. Bioelectron..

[49] Daggumati P., Matharu Z., Wang L., Seker E. (2015). Biofouling-Resilient
Nanoporous Gold Electrodes for DNA Sensing. Anal. Chem..

[50] Lord M. S., Foss M., Besenbacher F. (2010). Influence
of nanoscale surface topography
on protein adsorption and cellular response. Nano Today.

[51] Karasu T., Çalışır F., Pişkin S., Özgür E., Armutcu C., Çorman M. E., Uzun L. (2024). An intriguing future is approaching: Artificial intelligence meets
molecularly imprinted polymers. Journal of Pharmaceutical
and Biomedical Analysis Open.

[52] Wang M., Cetó X., del Valle M. (2022). A Sensor Array
Based on Molecularly
Imprinted Polymers and Machine Learning for the Analysis of Fluoroquinolone
Antibiotics. ACS Sensors.

[53] Dashtaki R. M., Dashtaki S. M., Heydari-Bafrooei E., Piran M. J. (2025). Enhancing the Predictive
Performance of Molecularly Imprinted Polymer-Based Electrochemical
Sensors Using a Stacking Regressor Ensemble of Machine Learning Models. ACS Sensors.

[54] Patois T., Lakard B., Monney S., Roizard X., Fievet P. (2011). Characterization
of the surface properties of polypyrrole films: Influence of electrodeposition
parameters. Synth. Met..

[55] Dantas H. B., Silva-Junior A. G., Silva N. L. C. L., Errachid A., Oliveira M. D. L., Andrade C. A. S. (2025). Genosensor
based on polypyrrole and
dendrimer-coated gold nanoparticles for human papillomavirus detection. Biochem. Eng. J..

[56] Cheng Q., Pavlinek V., Li C., Lengalova A., He Y., Saha P. (2006). Synthesis and structural properties of polypyrrole/nano-Y2O3
conducting composite. Appl. Surf. Sci..

[57] Gupta S., Acharya U., Pištěková H., Taboubi O., Morávková Z., Kašparová M., Humpolíček P., Bober P. (2021). Tuning the Conductivity,
Morphology, and Capacitance with Enhanced Antibacterial Properties
of Polypyrrole by Acriflavine Hydrochloride. ACS Applied Polymer Materials.

[58] Zhang Y., Xu A., Yu Y., Ye S., Zhao Z., Cao W., Zhang S., Qin Y. (2024). One-Step Fabrication
of Integrated
Graphene/Polypyrrole/Carbon Cloth Films for Supercapacitor Electrodes. Langmuir.

[59] Liu C., Xu Y., Huang B., Zhang W., Zhao X., Wang Y. (2024). UV-Curable
3D-Printable Microwave-Absorbing Material with a Sword-Sheath Structure
Based on Multiwalled Carbon Nanotube/Polypyrrole Nanotube/Fe3O4 Composites. Adv. Eng. Mater..

