# Supplementary material for: Low-Cost Polyphenol–Polypyrrole Molecularly Imprinted Sensor for Point-of-Care Alzheimer’s Detection
Source: ACS Sens. 2025 Aug 15;10(11):8435–46. doi: 10.1021/acssensors.5c01816 (PMC12671001; doi:10.1021/acssensors.5c01816)
Supplement: Supplementary file 1 [file se5c01816_si_001.pdf]

Supplementary Information

A Low-Cost Polyphenol-Polypyrrole Molecularly Imprinted Sensor for Point-of-Care Alzheimer's Detection

Ajith Mohan Arjun[1], Sudhaunsh Deshpande[1], Guoyi Liu[1,2], Daimei Miura[3,4], Krzysztof Pawlak [5], Tokuda Takahiko [6], Makoto Higuchi[6], Miyu Matsumoto[3], Tomoko Umemura[3], Kaori Tsukakoshi[3], Sanjiv Sharma[1*]

[1]David Price Evans Global Health and Infectious Diseases Group, Pharmacology & Therapeutics, Institute of Systems, Institute of Systems, Molecular and Integrative Biology, University of Liverpool, Crown Street, Liverpool L69 7BE, United Kingdom.

[2]Key Laboratory of Optoelectronic Technology & Systems (Chongqing University), Chongqing 400044, China.

[3]Department of Biotechnology and Life Science, Tokyo University of Agriculture and Technology, 2-24-16, Naka-cho, Koganei, Tokyo 184-8588, Japan.

[4]Institute of Global Innovation Research, Tokyo University of Agriculture and Technology, 3-8-1 Harumi-cho, Fuchu, Tokyo 183-8538, Japan

[5]Materials Innovation Factory, University of Liverpool, 51 Oxford Street, Liverpool L7 3NY.

[6]Advanced Neuroimaging Center, Institute for Quantum Medical Science, National Institutes for Quantum Science and Technology (QST), 4-9-1 Anagawa, Inage-ku, Chiba-shi, Chiba, 263-8555, Japan.

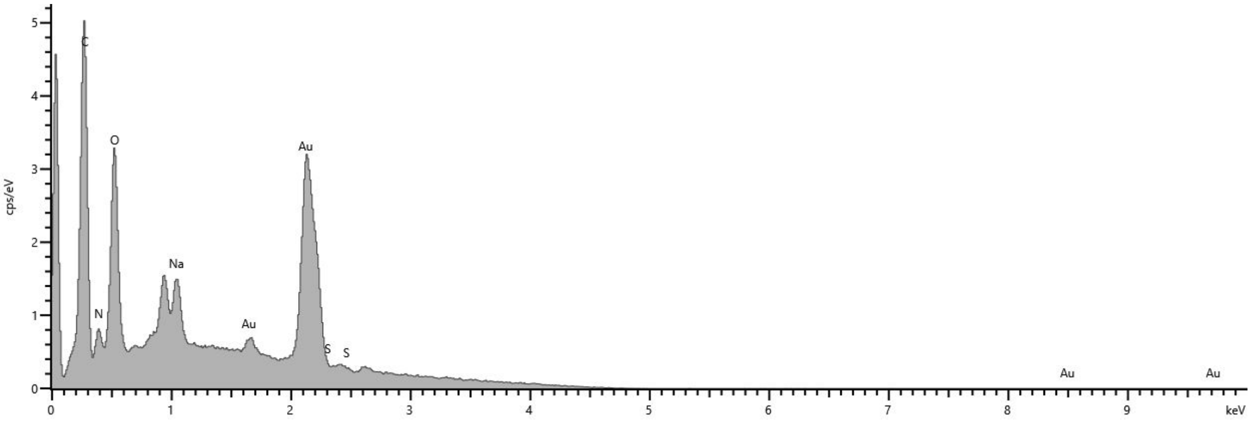

Figure S1: EDS spectra of the pPhR-pPY composite

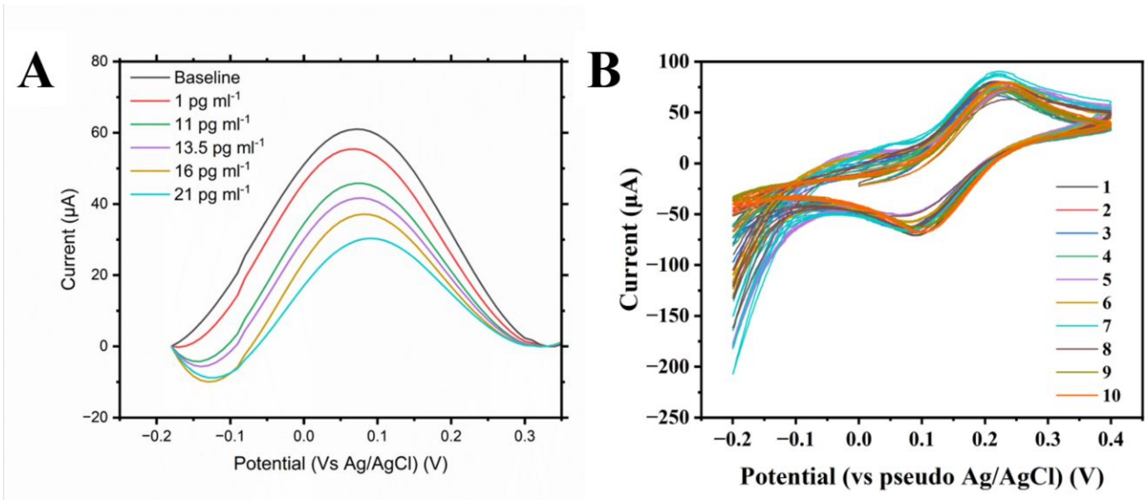

Figure S2: (A) DPVs of pPHR carried out in PBS. (B) CVs of multiple sensors in ferricyanide solution.

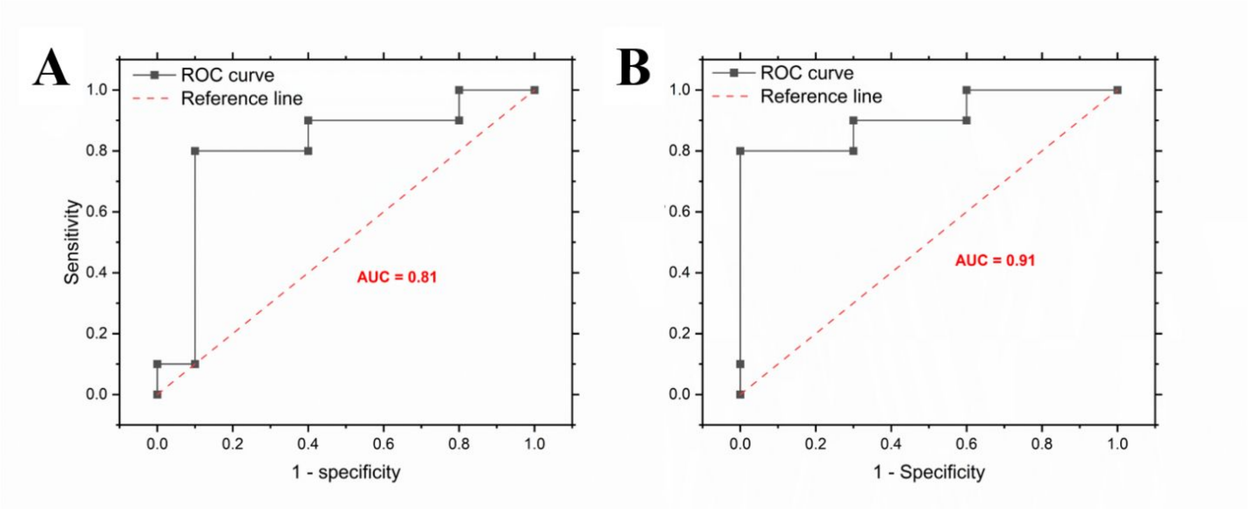

Figure S3: ROC curves of the (A) pPHR sensor and (B) SIMOA in real samples

Table S1. Classification Model Performance Metrics on Test Dataset

**Metric**	**Value**
F1 Score	1.00
Accuracy	1.00
Precision	1.00
Recall	1.00

Description: Classification was performed using a supervised learning pipeline on a dataset derived from 2,750 spiked plasma and serum samples. The feature set was extracted using a chemometric algorithm from DPV curves. A 75:25 train–test split was used, and 5-fold cross-validation confirmed robustness. All reported values are based on test set performance.

Table S2. Regression Model Performance Metrics on Test Dataset

Metric	Value
Mean Absolute Error (MAE)	0.35 pg.mL^{-1}
Mean Squared Error (MSE)	0.66
Root Mean Squared Error (RMSE)	0.81
R^2 Score	0.96

Description: Regression modelling was used to quantify p-Tau181 concentrations based on extracted electrochemical features. The same dataset and validation strategy as in Table S1 were employed.

Table S4. Intra- and Inter-Assay Variance for Clinical Samples (pPhR-pPy MIP vs. pPy Control)

Patient Type	Variance (pPhR)	Variance (pPy)
AD	0.02905	0.00543
AD	0.13188	0.00340
AD	0.03750	0.01669
AD	0.01440	0.03049
AD	1.42654	0.00757
AD	0.08586	0.02917
AD	0.00314	0.00109
AD	0.16602	0.07476
AD	0.01439	0.00516
AD	0.01869	0.00043
HC	0.09836	N/V
HC	0.02536	0.01449
HC	0.05687	0.08827
HC	0.02854	0.00453
HC	0.03946	0.05630
HC	N/V	N/V
HC	0.00774	0.00617
HC	0.14187	0.01003
HC	0.06247	0.26383
HC	0.08449	0.08649

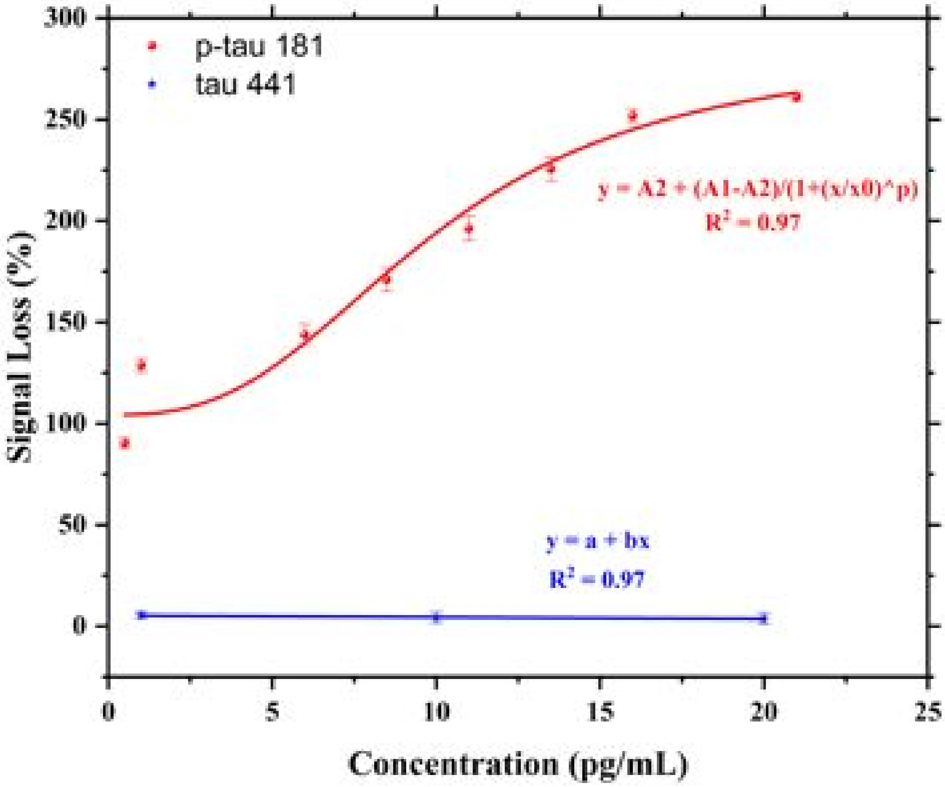

Figure S4: Dose–response curves comparing the electrochemical signal generated by p-Tau181 and Tau441 recombinant proteins using the pPhR-pPy MIP sensor in phosphate-buffered saline (PBS). The response to p-Tau181 was significantly higher than that to Tau441 at equivalent concentrations, indicating favorable selectivity of the molecularly imprinted layer toward the phosphorylated isoform.

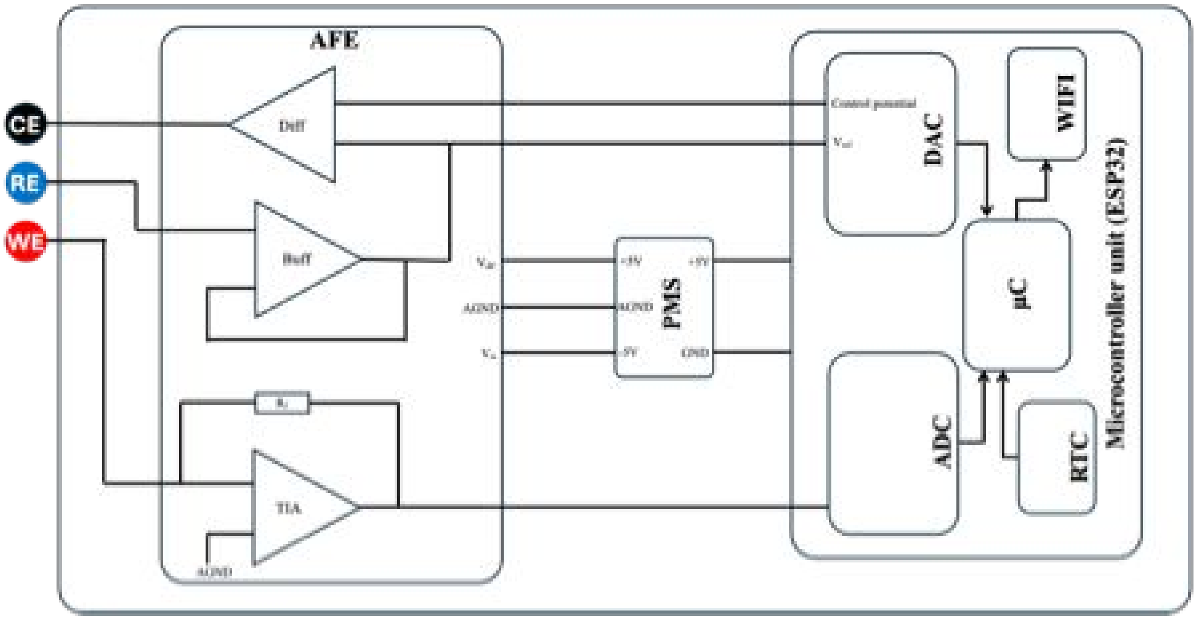

Figure S5: Functional block diagram of the custom-designed handheld potentiostat used for real-time electrochemical sensing. The analog front end (AFE) is built around the LM324N quad operational amplifier and includes a differential amplifier (Diff), buffer amplifier (Buff), and transimpedance amplifier (TIA) for signal conditioning from the three-electrode setup (working electrode, WE; reference electrode, RE; counter electrode, CE). The power management system (PMS) provides isolated ±5 V rails and analog ground using a TEA-10505 DC–DC converter. The microcontroller unit (ESP32) includes a digital-to-analog converter (DAC) for potential control, an analog-to-digital converter (ADC) for current acquisition, a real-time clock (RTC) for timing precision, and Wi-Fi for wireless data transfer. Acquired signals are processed into JSON format and transmitted to a web app for machine learning analysis. A GPIO-controlled mechanical relay enables electrode multiplexing between WE1 and WE2.

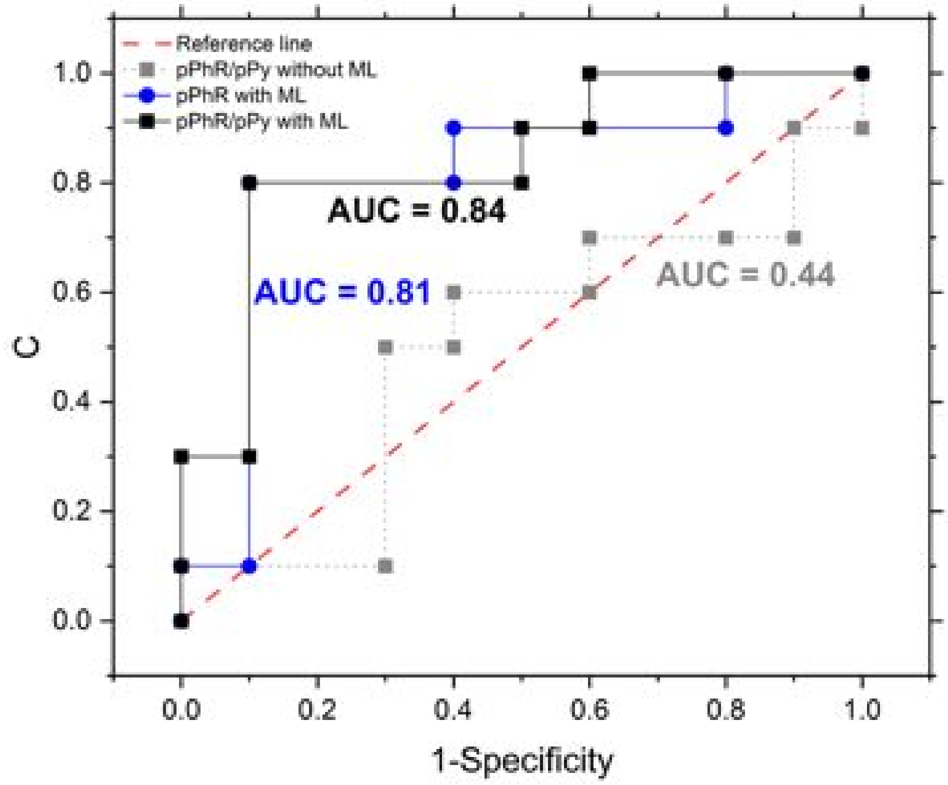

Figure S6: Receiver Operating Characteristic (ROC) curves comparing the classification performance of ML-assisted versus non-ML approaches. The pPhR/pPy MIP sensor with ML (black) achieved the highest area under the curve (AUC = 0.84), followed by pPhR with ML (blue, AUC = 0.81). In contrast, the non-ML approach applied to pPhR/pPy sensor responses (grey) resulted in poor classification (AUC = 0.44), comparable to random chance (red dashed reference line). These results demonstrate the advantage of machine learning in extracting meaningful patterns from complex biosensing data.

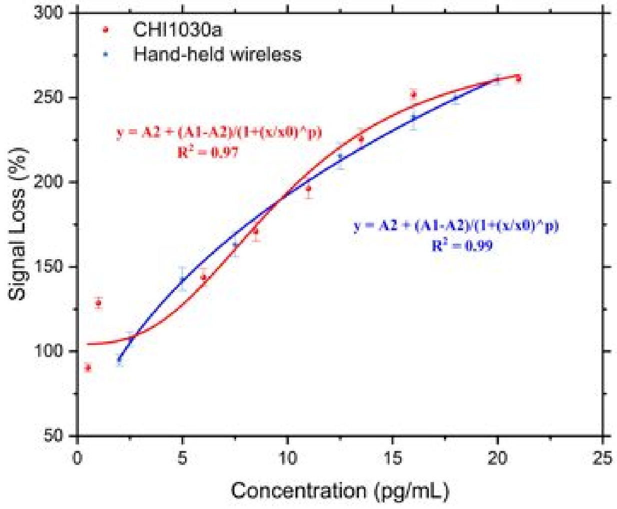

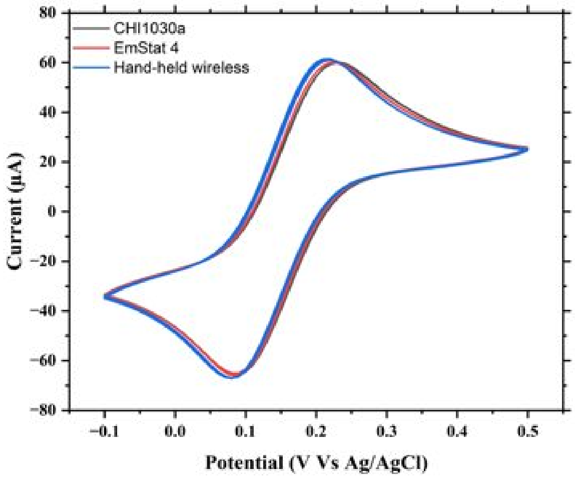

Figure S7: Validation of the handheld wireless potentiostat against commercial instruments.

(A) Dose-response calibration curves for p-Tau181 detection using the handheld potentiostat (blue) and the CHI1030a benchtop system (red) show excellent agreement, with nonlinear regression fits yielding $R^2 = 0.99$ and $R^2 = 0.97$, respectively.

(B) Cyclic voltammetry (CV) profiles of 5 mM $[Fe(CN)_6]^{3-/4-}$ in 0.1 M KCl using the handheld potentiostat (blue), CHI1030a (black), and EmStat4 (red), demonstrating comparable signal fidelity and electrochemical performance across platforms.
